# Adult idiopathic cholestasis: a condition more common in the Canadian Inuit?

**DOI:** 10.1080/22423982.2017.1388104

**Published:** 2017-10-15

**Authors:** Gerald Y. Minuk, Galia Pollock, Julia Uhanova

**Affiliations:** ^a^ Section of Hepatology, Department of Medicine, College of Medicine, University of Manitoba, Winnipeg, Canada; ^b^ Department of Pharmacology and Therapeutics, College of Medicine, University of Manitoba, Winnipeg, Canada

**Keywords:** Adult idiopathic cholestasis, cholangitis, cholestasis, chronic liver disease, cryptogenic cirrhosis, Inuit

## Abstract

Despite extensive investigations, some patients have no identifiable cause for their cholestatic liver enzyme abnormalities. The aim of this study was to document the clinical, laboratory, radiologic and histologic features of adult patients with idiopathic cholestasis (AIC). A computerised database of referred patients to a tertiary care hospital outpatient department for assessment of hepatobiliary disorders between 2005 and 2015 was employed to identify and describe features associated with AIC. Of 6,560 patient referrals, sufficient documentation to warrant a diagnosis of AIC was present in 17 (0.26%) cases. Of the 17, a disproportionate number were Canadian Inuit (7/60, 12% Inuit referrals vs. 10/6,500, 0.16% non-Inuit referrals, p<0.0001). The median age of the 17 subjects was 57 years and nine (53%) were female. Clinical and/or laboratory evidence of autoimmune disorders was present in six (35%) cases. Clinical features of hepatic decompensation, radiologic findings in keeping with cirrhosis and histologic confirmation of cirrhosis were present in 47%, 31% and 42% of individuals, respectively. There were no significant improvements in cholestatic liver enzymes and function tests in those treated with ursodiol and/or immunomodulants (n=7) compared to those left untreated (n=10). In conclusion, AIC is a rare condition diagnosed by exclusion. It appears to be more common in the Canadian Inuit population and those with autoimmune disorders. Advanced liver disease is a frequent finding at presentation. Intervention with ursodiol and/or immunomodulants does not appear to be of therapeutic value.

## Introduction

Clinical observations at our centre suggested a disproportionate number of Canadian Inuit patients were being referred for investigation of cholestatic liver enzyme abnormalities. In many cases, despite careful clinical evaluations, extensive laboratory testing, abdominal imaging and histologic analyses, a cause for the cholestasis could not be identified. Moreover, in some cases, features of hepatic decompensation were present, suggesting a progressive form of liver disease. To determine whether this cohort of Inuit patients with what we tentatively designated “Adult Idiopathic Cholestasis” (AIC) differed from that of non-Inuit patients with unexplained cholestasis, we compared the demographic, clinical, biochemical, serologic, radiologic and histologic features of the two cohorts.

## Methods

### Patient selection

The study consisted of accessing data from a computerised database supplemented by chart reviews of adult patients with unexplained chronic cholestasis referred to the Liver Diseases Outpatient Program (LDOP) at the Health Sciences Centre (HSC) in Winnipeg, Manitoba, Canada, for further investigations and/or management of their liver disease. The HSC is a tertiary care centre serving a population of ~1.5 million inhabitants of the provinces and territories of Manitoba, Northern Ontario and Nunavut.

Inuit patients were identified by the University of Manitoba’s Northern Medical Unit as being of Inuit ethnicity and referred to the LDOP for further evaluation of their liver disease between the years 2005 and 2015. The same time frame and database were employed to identify non-Inuit subjects referred to the LDOP with unexplained cholestatic liver disease.

### Investigations

The following data were collected where available: patient age, gender, body mass index (BMI), symptoms of fatigue, pruritus, presence or history of autoimmune disorders, clinical signs of hepatic decompensation (hepatic encephalopathy, portal hypertensive bleeding, jaundice or fluid retention), complete blood counts (CBC), serum alanine aminotransferase (ALT), aspartate aminotransferase (AST), alkaline phosphatase (ALP), gamma-glutamyl transferase (GGT), total bilirubin (T.Bili), direct bilirubin (D.Bili), albumin, international ratio of prothrombin times (INR), creatinine, immunoglobulin (IgA, IgG, IgM and IgG4), C_3_, C_4_, antibodies to nuclear (ANA), smooth muscle (SMA), mitochondria (AMA), peripheral (P-ANCA) and cytoplasmic (C-ANCA) nuclear cytochrome antigens, ceruloplasmin, cholesterol, ferritin, alpha 1-antitrypsin, alpha fetoprotein, CA 19–9 and carcinoembryonic antigen (CEA) levels. Hepatitis B surface antigen (HBsAg) and antibodies to hepatitis C virus (anti-HCV) were also documented. Radiologic imaging consisted of abdominal ultrasounds, computerised tomography (CT), magnetic resonance imaging (MRI-MRPC) and chest x-rays (CXR). When performed, liver biopsies were obtained within 6 months of the patient’s initial visit and prior to therapy.

### Diagnosis of AIC

Patients were considered to have AIC if the serum ALP levels exceeded 1.5 times the upper limit of normal, with an accompanying increase in GGT values and minimal elevations in serum aminotransferase values on at least two occasions 6 months apart and common causes of cholestasis had been excluded. The latter included (1) negative abdominal imaging for pathologic dilation of the biliary tract, choledocholithiasis, strictures, tumours, flukes and space occupying lesions within the liver, (2) an AMA titre measured on at least two occasions, with both results being negative or with titres less than 1:40, (3) negative serology for HBsAg and anti-HCV, (4) negative CXR for signs of tuberculosis, sarcoidosis and lymphoma, (5) no history of medications commonly implicated as causes of cholestasis being introduced within the previous 6 months, (6) no evidence of extrahepatic tumours or haematologic malignancies and, when available, (7) liver histology negative for florid duct lesions, onion skin fibrosis and a plasma cell infiltrate with greater than 10% of plasma cells staining positive for IgG4 immunoglobulin.

### Follow-up

In general, repeat clinical assessments and follow-up testing were performed every 6 months or earlier if clinically indicated. Treatments with ursodeoxycholic acid and/or immunosuppressive agents were recorded.

### Statistics

Univariate analysis included t-tests for continuous variables and Fisher’s exact tests for categorical variables, due to small sample sizes (i.e. n≤5). P-values less than 0.05 were considered significant. Results are provided as the medians and interquartile ranges (IQR) for the continuous variables and the proportions (%) for the categorical variables. Statistical analysis was conducted using non-parametric techniques. Wilcoxon Signed Rank Tests were conducted in order to test the changes in selected liver biochemistry tests over time for treated and untreated AIC patients. Similarly, Mann Whitney U-tests were conducted in order to compare the change in treated patients vs. the change in untreated patients. P-values less than 0.05 were considered significant.

## Results

A total of 65 adult Inuit patients had been referred to the LDOP for further investigations of their liver disease between 2005 and 2015. Of these, 17 were diagnosed with non-alcoholic steatohepatitis, 14 autoimmune hepatitis, six unexplained hepatitis, three radiologically confirmed choledocholithiasis, three PBC, one IgG4 disease, one alcoholic hepatitis and one patient with Dilantin-induced liver disease. Additional exclusions consisted of four patients with incomplete charts, three as a result of serum ALP levels being <1.5 × ULN, two with elevated ALP levels but only documented on one occasion and three with no liver biochemical abnormalities. Thus, a total of seven (12%) referred Inuit subjects were designated AIC.

Of the non-Inuit subjects, 6,500 had been referred to the LDOP within the same time period. Of these, 2,620 were diagnosed with viral hepatitis, 2,604 non-alcohol induced fatty liver disease (NAFLD) or alcohol induced liver disease and 1,286 with various other causes of liver disease. Based on liver biochemistry in keeping with cholestatic liver disease and negative results for the tests outlined above, a total of 10 (0.16%) referred non-Inuit subjects were designated AIC.

Features of the Inuit, non-Inuit and combined patient cohorts are provided in Table 1. Although Inuit patients had higher platelet counts and lower total bilirubin, C_3_ and C_4_ levels, there were no significant differences in other demographic, clinical, biochemical, radiologic or histologic features between the two cohorts. In addition, the duration of follow-up was not significantly different in the two cohorts (medians of 7 (IQR = 1.5–13) and 3 (IQR = 1–6) years, respectively, p=0.28). Thus, in order to provide a more robust description of AIC, the two cohorts were combined for further analyses.

**Table 1.** Features of non-Inuit and Inuit patients with adult idiopathic cholestasis.

Variable	Non-Inuit (n=10)	Inuit (n=7)	Total (n=17)	p-value
*Demographics*				
Age	65 (38–77)	53 (46–62)	57 (42.5–70.5)	0.22
Gender (F:M)	5:5	4:3	9:8	1.00
BMI	27.2 (22.5–35.15)	36.3 (18–41.3)	29.6 (21.75–37.88)	0.77
*Characteristics*				
Fatigue	4/10 (40.0%)	3/7 (42.9%)	7/17 (41.2%)	1.00
Pruritus (initial visit)	1/10 (10.0%)	1/7 (14.3%)	2/17 (11.76%)	1.00
Pruritus (follow up)	5/10 (50.0%)	2/7 (28.6%)	7/17 (41.2%)	0.62
Decompensation*^a^*	6/10 (60%)	2/7 (28.6%)	8/17 (47.1%)	0.33
Autoimmune disorder*^b^*	1/10 (10%)	3/7 (42.9%)	4/17 (23.5%)	0.10
*Laboratory* *Median (IQR)*				
Hgb	131 (113.8–147.5)	124 (109–137)	125 (113.5–145)	0.54
WBC	6.9 (5.6–8.4)	6.8 (5.2–8.4)	6.8 (5.8–8.25)	0.91
Platelets	220.5 (139–325)	334 (273–481)	273 (191–393)	*0.05*
INR	1 (0.9–1.05)	0.9 (0.9–1.2)	0.9 (0.9–1.1)	0.69
ALT	68.5 (52.25–100.25)	47 (44–129)	68 (45–107.5)	0.87
AST	58 (41.25–78.25)	44 (30–73)	57 (36.5–74)	0.40
ALP	425 (210–634)	258 (200–514)	309 (207–534.5)	0.35
GGT	571.5 (132.25–706.25)	394 (228–636)	462 (190–694)	0.81
Albumin	39.5 (35.5–40.5)	33 (26.75–42)	39 (30.5–41.5)	0.55
Total Bilirubin	16 (9.5–100)	9 (6–10)	10 (7–21.5)	*0.024*
Conjugated Bilirubin	8 (3–127)	2.5 (1.75–4.25)	4 (2–10)	0.07
Ceruloplasmin	461 (324.25–667.25)	396 (n=1)	456 (331.5–650.0)	0.85
LDL	2.33 (2.19–3.8)	2.76 (1.3–3.52)	2.33 (2.19–3.59)	0.86
IgA	2.99 (1.77–4.69)	2.64 (2.07–3.73)	2.82 (1.91–3.82)	0.99
IgG	11.4 (9.25–14.25)	14 (12.8–19.6)	13.3 (11.125–17.35)	0.07
IgM	1.12 (0.78–3.31)	1.24 (0.86–2.92)	1.18 (0.87–2.76)	0.99
C3	1.49 (1.42–1.75)	1.095 (0.93–1.20)	1.24 (1.09–1.49)	*0.05*
C4	0.33 (0.31–0.34)	0.21 (0.115–0.245)	0.25 (0.19–0.33)	*0.05*
AMA	0/10 (0%)	0/7 (0%)	0/17 (0%)	
ANA	3/9 (33.3%)	1/6 (16.7%)	4/15 (26.7%)	0.60
SMA	2/8 (25.0%)	1/7 (14.3%)	3/15 (20.0%)	1.00
pANCA	0/4 (0%)	1/6 (16.7%)	1/10 (10%)	1.00
cANCA	0/4 (0%)	1/6 (16.7%)	1/10 (10%)	1.00
*Histology*				
Lymphocytic infiltration of portal tract	6/6 (100%)	3/6 (50%)	9/12 (75%)	0.18
Interface Hepatitis	0/6 (0%)	3/6 (50.0%)	3/12 (25.0%)	0.18
Granuloma	0/6 (0%)	1/6 (16.7%)	1/12 (8.3%)	1.00
Ductular proliferation	2/6 (33.3%)	0/6 (0%)	2/12 (16.67)	0.45
Ductopenia	1/6 (16.7%)	0/6 (0%)	1/12 (8.3%)	1.00
Fibrosis (stage 1–3)	3/6 (50.0%)	2/6 (33.3%)	5/12 (41.7%)	0.45
Cirrhosis	3/6 (50%)	2/6 (33.3%)	5/12 (41.7%)	1.00
*Treatment*				
Ursodiol	3/10 (30%)	0/7 (0%)	3/17 (17.6%)	0.23
Immunomodulant*^c^*	1/10 (10%)	1/7 (14.3%)	2/17 (11.8%)	1.00
Ursodiol + immunomodulant	0/10 (0%)	2/7 (28.6%)	2/17 (11.8%)	0.15
*Follow-up*				
Years since initial visit	7 (1.5–13)	3 (1–6)	4 (1.25–7)	0.28
Survival	10/10 (100%)	7/7 (100%)	17/17 (100%)	

The median age of all AIC patients was 57 years (IQR = 42.5–70.5) with an approximately equal gender distribution. Seven (42%) patients complained of fatigue and two (12%) pruritus. Five patients had physical findings of fluid retention (three with ascites and four peripheral oedema), three splenomegaly and one patient had a history of variceal bleeding. No patient had symptoms or signs of hepatic encephalopathy. Overall, 8/17 (47.1%) patients had clinical evidence suggestive of decompensated liver disease. A total of four (24%) subjects had what would be considered an autoimmune or connective tissue disorder: two (12%) rheumatoid arthritis, one (5.8%) psoriasis and one (5.8%) vitiligo.

Laboratory investigations revealed anaemia in nine (47%), leukopenia in three (17.7%) and thrombocytopenia in two (11.8%) patients. As per the study design, all patients had elevated serum ALP and GGT levels. Serum ALP levels were approximately 3-fold elevated beyond the laboratory’s upper limit of normal (median = 309 U/L, IQR = 207–534.5) and GGT values 16-fold elevated (median = 462 U/L, IQR = 190–694). Serum bilirubin levels, both total and conjugated, were elevated in 4/17 (23.5%) and 5/15 (33.3%) patients, with median values of 10 (IQR = 7–21.5) and 4 (2–10) µmol/L, respectively. Other biochemical markers of hepatic dysfunction such as low serum albumin levels were present in 5/16 (31.3%) (median = 39 g/L, IQR = 30.5–41.5) and high INR values in 2/15 (13.3%) (median = 0.9, IQR = 0.9–1.1) patients. Serum ceruloplasmin levels were elevated in 5/9 (55.6%) (median = 456 mg/L, IQR = 331.5–650) and IgM levels in 4/16 (25%) (median = 1.18 g/L, IQR = 0.87–2.76) patients. Plasma LDL levels were increased in 2/17 (28.6%) (median = 2.33 mmol/L, IQR = 2.19–3.59) patients. One of 14 (7.2%) patients had an elevated serum alpha fetoprotein and 2/12 (16.7%) elevated CEA and CA 19–9 levels, respectively. These values did not increase over time. Tests that often reflect the status of the immune system such as serum IgG, C_3_ and C_4_ levels were abnormal in 4/16 (25%), 1/7 (13.3%) and 1/7 (14.3%) patients, respectively. Autoantibodies including ANA, SMA, pANCA and cANCA were positive in 4/15 (27%), 3/15 (20%), 1/10 (10%) and 1/10 (10%) patients, respectively. One of 16 patients (6.25%) was positive for both ANA and SMA, and 1/10 (10%) positive for ANA and cANCA.

On radiologic imaging, 4/13 (31%) patients had what was reported as non-pathologic, mild common bile duct dilation (all with a history of a previous cholecystectomy and subsequently negative MRCP studies) and one patient had gallstones but no evidence of choledocholithiasis. Four of 13 (31%) had imaging features consistent with cirrhosis and/or portal hypertension (irregular liver edge, splenomegaly, oesophageal or gastric varices, ascites and/or reversal of portal venous flow).

Twelve patients (71%) underwent liver biopsies. Ductopenia was present in one (8.3%), lymphocytic infiltration of the portal tract in nine (75%), interface hepatitis in three (25%), portal granuloma in one (8.3%), ductular proliferation in two (16.7%), fibrosis (stages 1–3) in five (42%) and cirrhosis in five (42%) biopsies.

Median follow-up was 4 years (IQR = 1.25–7). At the end of this follow-up period, no additional patients complained of fatigue, but 5/15 (33%) previously non-pruritic patients developed pruritus. Thus, a total of seven (41.2%) patients were pruritic during the study period. During follow-up, a total of three (18%) subjects were treated with ursodiol (10–15 mg/kg) alone, two (11.8%) with an immunosuppressive agent (prednisone or methotrexate) alone, two (11.8%) with ursodiol plus an immunosuppressive agent (prednisone, methotrexate and/or azathioprine) and 10 (59%) left untreated. The results of the last follow-up liver enzyme and function tests in treated and untreated patients are provided in Table 2. Overall, serum ALP levels decreased by 38.5%, GGT 56.7%, total and direct bilirubin levels by 30% and 27.5%, respectively, while INR values increased by 11.1%. Of interest, the improvements in ALP levels were largely driven by results in the untreated cohort (Tables 3 and 4). In terms of comparative changes from baseline (delta), only improvements in serum albumin levels were significantly different (Δ treated = 7 versus Δ untreated = −2, p=0.038) (Table 5). However, these differences could also be explained by the untreated cohort having less advanced disease at baseline. None of the patients developed hepatocellular or cholangiocarcinoma and there were no deaths during the follow-up period.

**Table 2.** Changes in selected liver biochemistry tests over time (treated and untreated).

All patients (n=17)	Variable	Initial	Follow-up	Percentage change	p-value
	ALP	309 (207–534.5)	190 (126–345)	−38.5%	*0.009*
	GGT	462 (190–694)	200 (59–554)	−56.7%	0.12
	Albumin	39 (30.5–41.5)	38 (35.5–39.5)	−2.56%	0.93
	Total bilirubin	10 (7–21.5)	7 (6–11.5)	−30%	0.15
	Direct bilirubin	4 (2–10)	2.9 (2–5)	−27.5%	0.30
	INR	0.9 (0.9–1.1)	1.0 (0.9–1.0)	+11.1%	0.80

**Table 3.** Changes in selected liver biochemistry tests over time in untreated AIC patients (n=10).

Untreated patients (n=10)	Variable	Initial	Follow-up	Percentage change	p-value
	ALP	283.5 (196.5–524.25)	176 (129.5–325.5)	−37.9%	*0.049*
	GGT	376 (141.25–706.25)	191.5 (55.25–516.5)	−49.1%	0.29
	Albumin	40 (36–42)	37.5 (33.25–39.25)	−6.25%	0.20
	Total Bilirubin	10 (8.25–15.5)	6.5 (5.75–11.25)	−35%	0.29
	INR	1.0 (0.9–1.1)	1.0 (0.9–1.1)	0%	0.90

**Table 4.** Changes in selected liver biochemistry tests over time in treated AIC patients (n=7).

Treated patients (n=8)	Variable	Pre-treatment	Post-treatment	Percentage change	p-value
	ALP	378 (238–601)	212 (114–395)	−43.9%	0.12
	GGT	636 (250–686)	241 (54–585)	−62.1%	0.20
	Albumin	32 (28–40)	39 (37–43)	+21.9%	0.22
	Total Bilirubin	9 (6–280)	7 (6.4–15)	−22.2%	0.40
	INR	0.9 (0.9–1.05)	0.95 (0.9–1.0)	+5.55%	0.85

**Table 5.** Changes in selected liver biochemistry tests over time (delta treated and delta untreated).

All patients (n=17)	Variable	Treated	Untreated	Percentage difference	p-value
	Delta ALP	−168 [(−268)–(−74)]	−73.5 [(−207.75)–(−35.25)]	−56.25%	0.35
	Delta GGT	−153 [(−467)–(−105)]	−130.5 [(−210)–(34.5)]	−14.7%	0.59
	Delta Albumin	7 [(−1)–(11)]	−2 [(−4)–(−0.5)]	−128.6%	*0.038*
	Delta Total bilirubin	−2.1 [(−265)–(0.4)]	−3 [(−5.25)–(0.25)]	42.9%	0.66
	Delta Direct bilirubin	0.9 [(−0.1)–(0.1)]	−2 [(−3.75)–(−0.25)]	−322%	0.61
	Delta INR	0 [(−0.15)–(0.05)]	0 [(−0.1)–(0.1)]	0%	0.83

## Discussion

The results of this study serve to identify a previously undescribed hepatobiliary disorder that can be referred to as Adult Idiopathic Cholestasis (AIC). Although AIC must be considered a diagnosis by exclusion, certain features of the condition appear to be common and could provide insights with respect to the underlying aetiology. For example, there appears to be an ethnic predisposition to AIC in that according to our referral (albeit not population-based) data, the condition is 100 fold more common in the Canadian Inuit population. In addition, autoimmune or connective tissue comorbidities are not rare. Perhaps the most disturbing feature of AIC is the high prevalence of advanced liver disease and hepatic decompensation, suggesting a chronic, progressive disorder.

The true prevalence of AIC in liver disease patients has yet to be determined. Although only 17/6,560 (0.26%) referred patients were diagnosed with AIC, in many instances the data available were deemed insufficient to warrant establishing the diagnosis. Hence, the prevalence may be a significant underestimate. Alternatively, some patients may have had atypical presentations of common underlying cholestatic liver diseases, which would have resulted in an overestimate of AIC prevalence. That the study was performed in a tertiary care centre introduces the issue of referral bias, which would have further contributed to an inappropriately high prevalence rate.

Of the many causes of cholestasis in adults, the conditions that most resemble AIC and, hence, represent particular diagnostic challenges are: AMA negative PBC, small duct PSC (SD-PSC), autoimmune cholangitis and drug induced cholestasis. In the present study, AMA negative PBC was excluded if florid duct lesions were absent on liver biopsy [1]. For those patients who had not undergone liver biopsy, the diagnosis was deemed unlikely by the absence of the classic triad for AMA negative PBC: high serum cholesterol, ceruloplasmin and IgM levels [2]. Regarding SD-PSC, the absence of concomitant IBD, negative MRCP findings and lack of concentric fibrosis of bile ducts on liver biopsy argued against that condition [3]. Autoimmune cholangitis was excluded if serum AST values were not low, ANA titres not high and cholangitis not present on liver biopsy [4]. Finally, drug-induced liver disease was ruled out following a careful review of each patient’s medication list for recently introduced agents commonly associated with cholestasis [5]. Of note, none of the patients in the study admitted to taking herbs or alternative medications within 6 months prior to their initial presentation.

The aetiology of AIC remains to be determined. The large number of Inuit patients and the tendency for this ethnic population to have cholestatic liver enzyme abnormalities favours a genetic cause, but the advanced age of patients at presentation and absence of positive family histories for cholestasis argue against a genetic aetiology. The association with autoimmune disorders, prevalence of autoantibodies and predominantly lymphocytic cell infiltration on liver histology are in keeping with an autoimmune aetiology, but median serum IgG, C_3_ and C_4_ levels were not abnormal and fewer than 30% of patients had abnormal test values [6]. Moreover, previous reports have described high prevalences of autoimmune liver disorders in North American Aboriginals and, therefore, the positive autoimmune features could be explained by the disproportionate number of Inuit subjects within the study population [7]. Finally, a viral aetiology should be considered on the basis of the lymphocytic infiltrates and, to some extent, presence of autoimmune antibodies [8]. However, of the two viruses most often associated with cholestatic liver disease; HCV and CMV, serology for both were negative and CMV infections of the liver tend not to persist beyond 6 months in immune competent hosts [9]. Nonetheless, other viral pathogens such as betaretroviruses and Reovirus serotype 3 which have been implicated in the pathogenesis of human cholestatic disorders should be considered and excluded in future studies [10,11].

According to clinical, radiologic and histologic findings, the majority of AIC patients have advanced disease at presentation. Not knowing the date of onset of the condition, this finding could be interpreted as indicating that AIC is a rapidly progressive disorder or, alternatively, associated with a prolonged subclinical phase of illness. Referral bias for Inuit patients from remote regions of Northern Canada might have also contributed to this finding. Notwithstanding these considerations, because AIC can be associated with advanced liver disease and hepatic decompensation, empiric treatment with ursodiol and/or immunosuppressive agents was utilised. Although in the small cohort of treated patients (n=7) treatment was not harmful, most biochemical features of the disease were not improved compared to baseline values and untreated patients.

There are many limitations to this study that warrant emphasis. First, the number of subjects was small and, therefore, type 1 and 2 statistical errors are likely. Second, as is often the case with studies employing retrospective study designs, not all data fields were complete and, in some instances, whole charts were not available. Third, other potentially informative tests such as genetic testing for multidrug resistance (MDR) protein mutations were not performed [12]. Fourth, long-term follow-up data were not available. Finally, in the absence of previous reports describing AIC or an alternatively labelled disorder, comparative analyses could not be performed to determine whether this cohort of subjects differ from AIC or AIC-like patients in other centres and geographic regions.

In conclusion, this report describes a previously undescribed chronic, cholestatic liver disease, AIC, which appears to be more common in the Inuit peoples and frequently associated with immune-mediated disorders and advanced liver disease. Further research is required to identify the aetiology, define the natural history and develop effective therapy for this condition.
